# Retrospective analysis of extra-articular distal humerus shaft fractures treated with the use of pre-contoured lateral column metaphyseal LCP by triceps-sparing posterolateral approach

**DOI:** 10.1007/s11751-016-0270-6

**Published:** 2016-11-03

**Authors:** Yatinder Kharbanda, Yashwant Singh Tanwar, Vishal Srivastava, Vikas Birla, Ashok Rajput, Ramsagar Pandit

**Affiliations:** 1 Department of Orthopedics, Apollo Hospital, HNo299, Pocket B, DDA Flats, Sarita Vihar, New Delhi, Delhi 110076 India; 2 Department of Orthopedics, Dr. RML Hospital and PGIMER, New Delhi, Delhi 110001 India

**Keywords:** Distal humerus fracture, Extra-articular distal humerus LCP, Posterolateral approach humerus

## Abstract

Management of extra-articular distal humerus fractures presents a challenge to the treating surgeon due to the complex anatomy of the distal part of the humerus and complicated fracture morphology. Although surgical treatment has shown to provide a more stable reduction and alignment and predictable return to function, it has been associated with complications like iatrogenic radial nerve palsy, infection, non-union and Implant failure. We in the present series retrospectively analysed 20 patients with extra-articular distal humerus shaft fractures surgically treated using the extra-articular distal humeral locking plate approached by the triceps-sparing posterolateral approach. The outcome was assessed using the DASH score, range of motion at the elbow and the time to union. The mean time to radiographic fracture union was 12 weeks.

## Introduction

Extra-articular fractures of the distal humeral shaft are relatively rare injuries and have been in the limelight owing to a higher incidence of radial nerve injuries, as well as the dilemmas surrounding their management [1, 2]. Both conservative and surgical treatment options exist for these fractures, with the ideal treatment still being debatable. Bracing has been an acceptable option for humeral shaft fractures; however, in the distal third of the humerus in adults it can cause problems owing to difficulty in controlling angulation. Sarmiento reported his results of functional bracing for comminuted extra-articular fractures of the distal third humerus. There was varus deformity averaging 9 degrees in 81% of patients, but loss of range of movement was minimal and functional results were good [3]. However, O`Driscoll et al. [4] showed that cubitus varus deformity secondary to supracondylar malunion or congenital deformity of the distal part of the humerus may not always be a benign condition and may have important long-term clinical implications including tardy posterolateral instability.

**Fig. 1:**
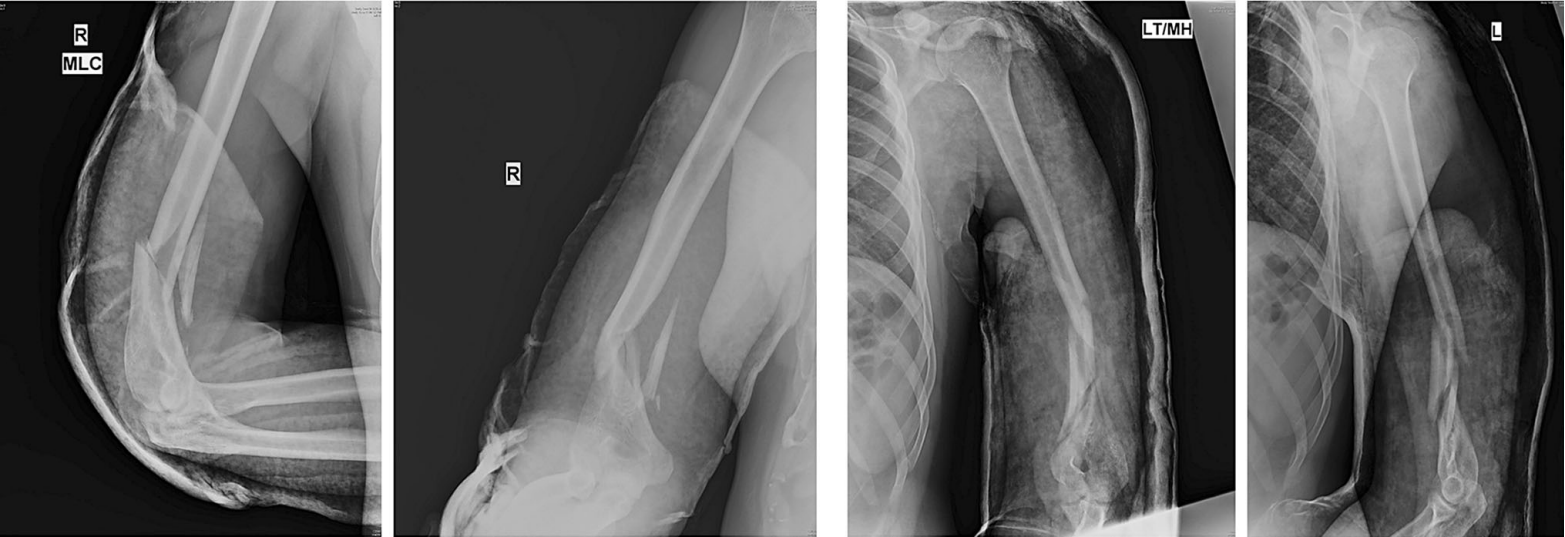
Showing AP and lateral views of X-rays with low distal humeral “extra articular” fracture

Although surgical treatment seems to provide a more reliable and predictable alignment and potentially quicker return of function, iatrogenic radial nerve palsy is a cause of major concern [5]. If the decision to proceed to surgical intervention has been made, then plate osteosynthesis is the usual standard option [6]. The classical teaching for fixation of a humeral shaft fracture has been with a narrow/broad 4.5 mm low-contact dynamic compression plate, purchasing a minimum of eight cortices (i.e. 4 screws) on either side of the fracture zone or at least six cortices (3 screws) on either side if a lag screw has been used [6]. This, however, becomes difficult to achieve in distal humeral shaft fractures owing to the limited space available distally, as well as the curved shape of the distal humerus when approaching anteriorly and the presence of the olecranon fossa posteriorly (Fig. 1). Double-column plating using two 3.5-mm plates in orthogonal or parallel patterns is another option [7], but it requires greater soft tissue stripping and exposure, leading to a potentially higher non-union and infection rate and elbow stiffness reported in some series [5, 8].

In the present retrospective case series, we present our clinical experience with use of a single column pre-contoured extra-articular distal humeral locking compression plate (J plate Synthes, Solothurn, Switzerland) for treatment of extra-articular distal humeral fractures. It was a retrospective study aimed to evaluate the clinical and radiographic results after fixation of fractures of the distal humerus shaft with this single column system.

## Materials and methods

### Implant

**Fig. 2:**
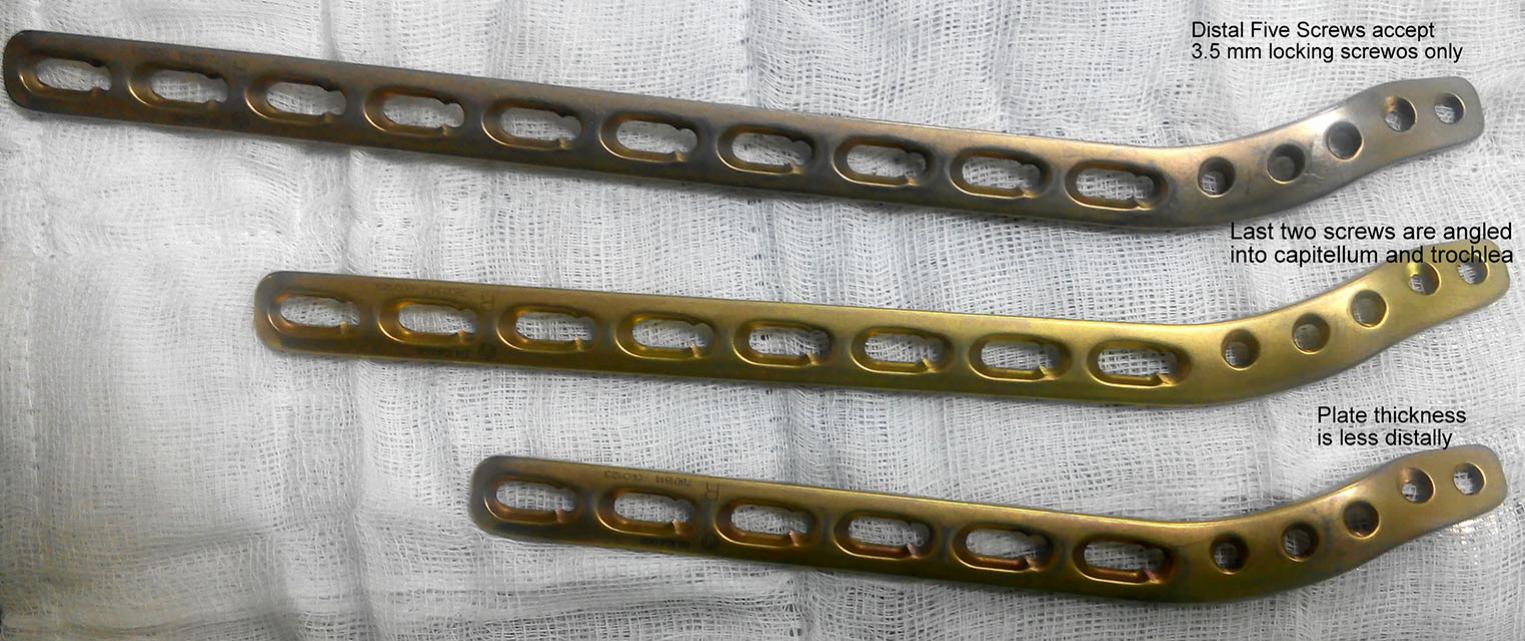
Extra-articular distal humerus plate

3.5-mm LCP (Locking Compression Plate) extra-articular distal humerus plate (AO Synthes) is an anatomically shaped and angular stable fixation system for extra-articular fractures of the distal humerus. Distally, the plate accepts five 3.5-mm locking screws and is tapered to minimize soft tissue irritation and the screw hole density is greater to allow larger number of screws to be placed in the distal fragment (Fig. 2). The two most distal screw holes are angled towards the capitellum and trochlea, which allows longer locking screws to be placed distally. Proximally, the thickness of the plate is based on LCP 4.5/5.0, narrow and has combi-holes. Locking screws create a fixed-angle construct, providing angular stability, whereas the combi-holes can be used to provide inter-fragmentary or dynamic axial compression. As the plates are anatomically contoured, there are different plates for the right and left sides and it is available from 4 hole (122 mm) to 14 (302 mm) hole length.

### Patients

Between Sept 2010 to Feb 2013, 20 patients with metaphyseal extra-articular distal humerus fractures—AO Type 12 A/B/C—were treated at our institution using the EADHP (Table 1). Inclusion criteria for the patients were: fractures of the distal humeral shaft which could not be fixed with conventional LCDCP’s with minimum of six/eight cortices distally, age >18 years, closed fractures of the distal humeral shaft, with or without radial nerve palsy, recent fractures and non-unions. Patients who did not satisfy these inclusion criteria were not included in the study. All the surgeries were performed by the same senior author (YK) at one institution only.

**Table 1:** Showing the different variables which were observed

Sr no.	Age	Sex	Mode of injury	Radial nerve	Time interval between injury and surgery in days	Associated injuries	Follow-up duration in months	Dash score at 1 year	Time to union in weeks	Elbow flexion	Elbow pronation supination	AO type	Number of lag screws	Plate length combi-holes	Proximal fixation	Distal fixation
1	31	F	Fall	Intact	1	Nil	24	14.2	12	0–130	85/85	12B2	2	8 Hole	3	5
2	42	M	Fall	Intact	2	Nil	15	13.3	16	0–140	80/80	12 A2	1	8 Hole	3	3
3	38	M	RTA	Intact	1	Ipsilateral shaft radius and ulna	18	15	16	0–120	80/75	12C1	3	8 Hole	3	5
4	56	F	RTA	Intact	4	Nil	38	18.3	18	0–135	90/85	12C1	3	10 Hole	3	5
5	62	M	Fall	Intact	2	Ipsilateral radius fracture	30	23.3	15	0–125	80/85	12B1	3	8 Hole	4	5
6	50	M	Fall	Neuropraxia	1	Nil	22	30	12	5–120	75/80	12C1	5	10 Hole	4	6
7	44	M	RTA	Intact	120	Nil	29	18.3	16	0–130	75/75	12B1	2	8 Hole	3	5
8	48	F	RTA	Intact	3	Nil	40	17.5	12	0–135	80/80	12B1	3	8 Hole	4	5
9	39	M	Fall	Intact	5	Ipsilateral tibia fracture	12	18.3	12	0–120	80/85	12A1	2	8 Hole	3	5
10	54	F	RTA	Intact	2	Nil	20	20	12	0–120	85/90	12C1	3	8 Hole	3	5
11	49	F	RTA	Intact	1	Nil	17	19.2	16	0–115	85/85	12B1	2	8 Hole	3	5
12	56	M	RTA	Neuropraxia	1	Nil	28	31.7	16	0–110	75/80	12B1	3	8 hole	3	6
13	37	M	Fall	Intact	2	Nil	33	15.8	10	0–130	90/90	12A1	3	8 Hole	3	5
14	33	M	RTA	Intact	2	Nil	42	15	12	0–130	90/90	12B1	2	8 Hole	3	5
15	52	M	Fall	Intact	1	Nil	22	14.2	18	0–120	85/90	12B1	3	8 Hole	3	5
16	46	M	Fall	Intact	2	Nil	21	12.5	12	0–125	90/90	12B2	2	8 Hole	3	5
17	40	F	RTA	Intact	90	Nil	18	16.7	16	0–115	85/85	12B1	3	10 Hole	4	5
18	35	F	RTA	Intact	2	Nil	32	15	12	0–130	90/90	12A1	2	8 Hole	3	5
19	37	M	Fall	Intact	1	Nil	36	11.7	12	0–135	90/90	12A2	1	8 Hole	3	5
20	32	M	RTA	Intact	1	Nil	15	12.5	12	0–125	85/80	12B1	2	8 Hole	4	5

Clinical outcome was assessed using Disabilities of the Arm, Shoulder and Hand (DASH) score and the range of motion of the elbow joint for each patient. The union was assessed clinically and radiologically; clinically by absence of pain and tenderness on palpation and range of motion at elbow joint, ability to perform activities of daily living without pain. Anteroposterior and lateral radiographs were done, and the healing progress of the distal humerus fracture was assessed. Union was defined by the absence of fracture line or bridging of the fracture site on at least 3 of the 4 cortices and the absence of implant loosening or failure.

### Surgical technique

**Fig. 3:**
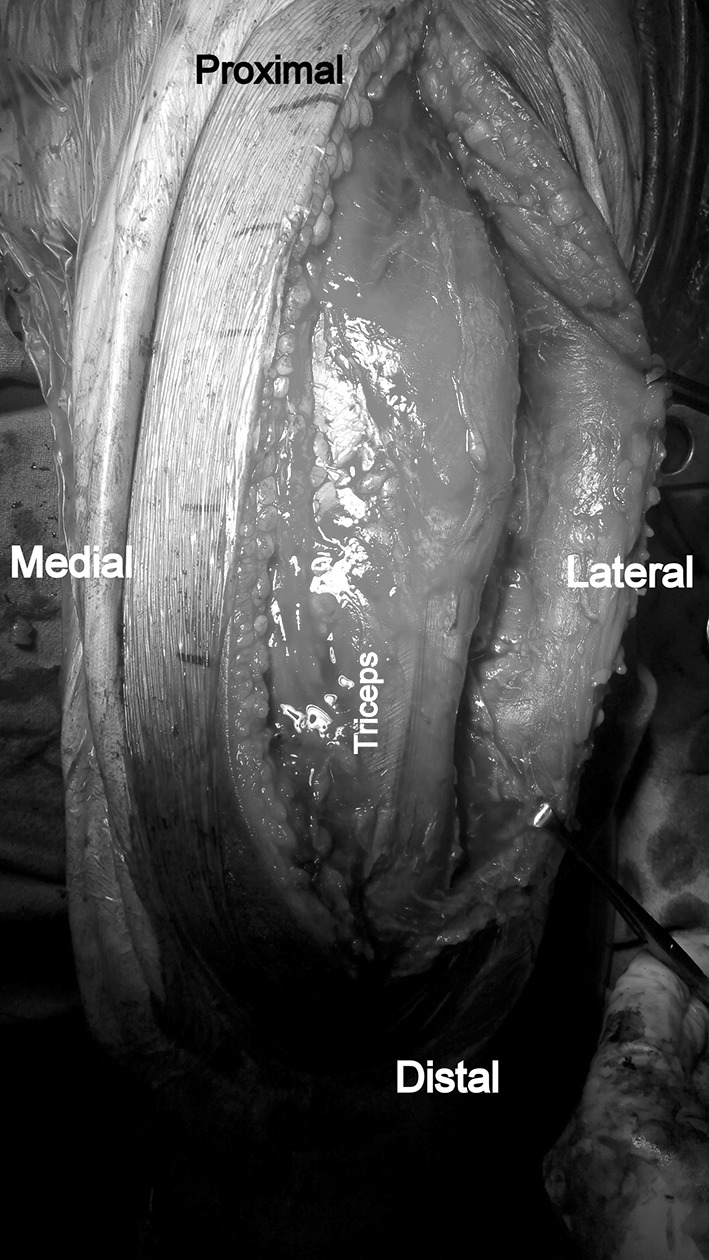
Midline skin incision and elevation of full thickness lateral flap

**Fig. 4:**
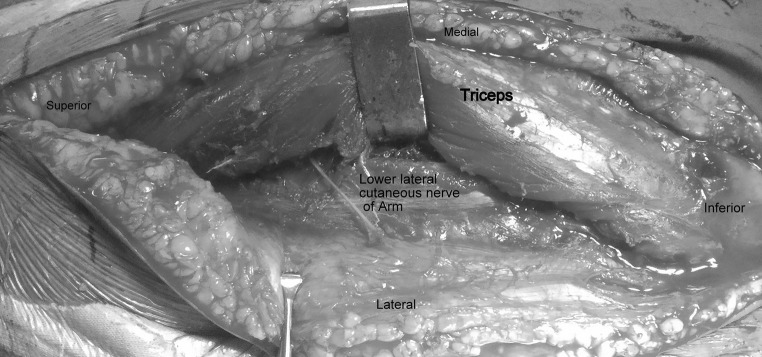
Lower lateral cutaneous nerve of the arm which can be traced proximally to the radial nerve

**Fig. 5:**
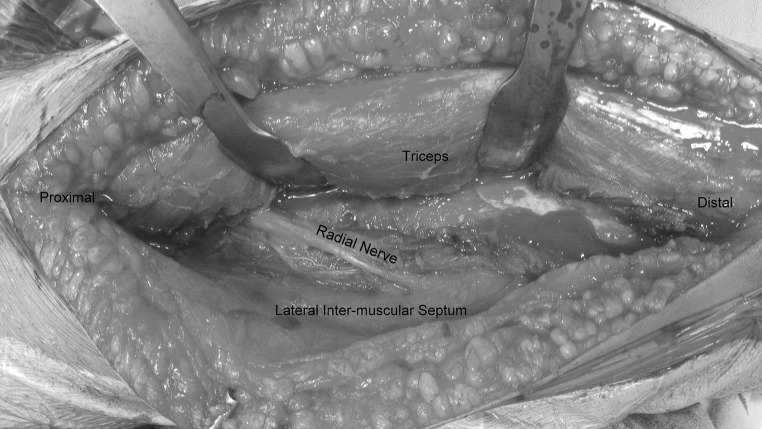
Elevation of the triceps from the lateral inter-muscular septum and radial nerve dissection

**Fig. 6:**
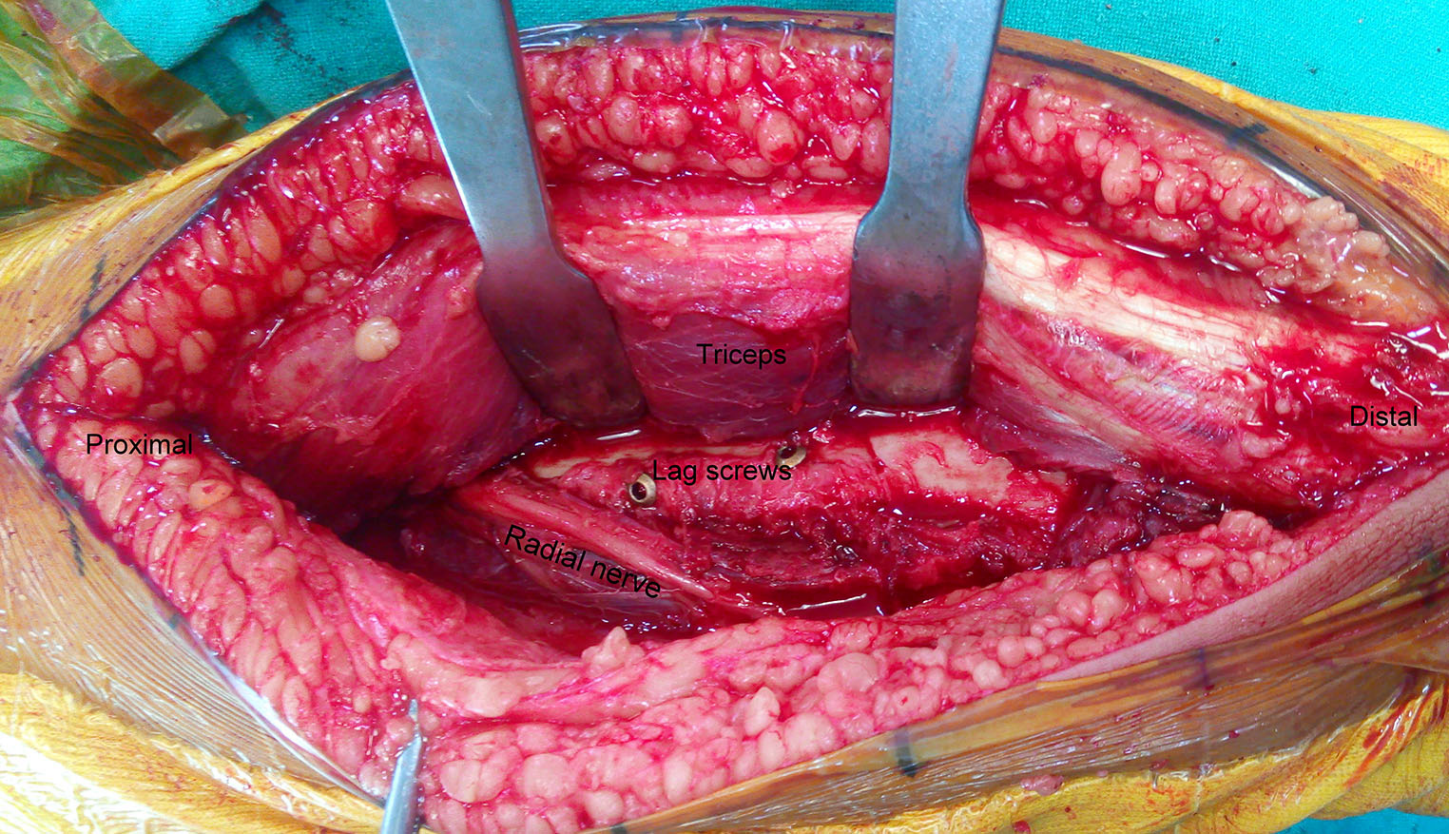
Lag screw fixation

**Fig. 7:**
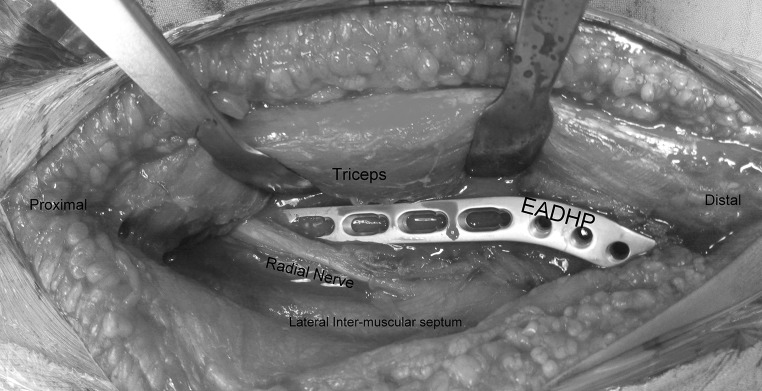
Placement of plate over lateral column, note that the medial column is not dissected at all

Patient is placed in the lateral position under general anaesthesia, with the arm hanging by the side. A triceps-reflecting posterolateral approach of Gerwin et al. [9] is utilized to expose the fracture site. After performing a midline skin incision on the posterior aspect of arm, full thickness flaps are developed on the lateral side (Fig. 3). On the lateral side, using blunt dissection, the lower lateral cutaneous nerve of the arm is identified and its origin traced to the radial nerve (Fig. 4). The triceps is elevated from the lateral inter-muscular septum and the lateral supracondylar ridge, and the radial nerve is then carefully dissected (Fig. 5). After adequate fracture visualization, reduction clamps are used to reduce the fracture fragments. Provisional fixation is achieved with K wires, and lag screws are used wherever possible to increase the strength of the construct and achieve adequate compression in spiral fractures (Fig. 6). Finally, the Synthes TM extra-articular distal humerus plate is applied over the posterior surface of humeral shaft and fixed with locking screws distally and a combination of cortical and locking screws proximally. The plate is positioned so that its shaft portion is located centrally on the posterior aspect of the humerus, while the distal end curved along the posterior aspect of the lateral column (Fig. 7). Plate bending is required in some cases for better seating of the plate to the bone surface. Post-operatively, the patient is placed in a soft dressing and arm pouch sling and early range of motion of the elbow, wrist and shoulder is started.

## Results

**Fig. 8:**
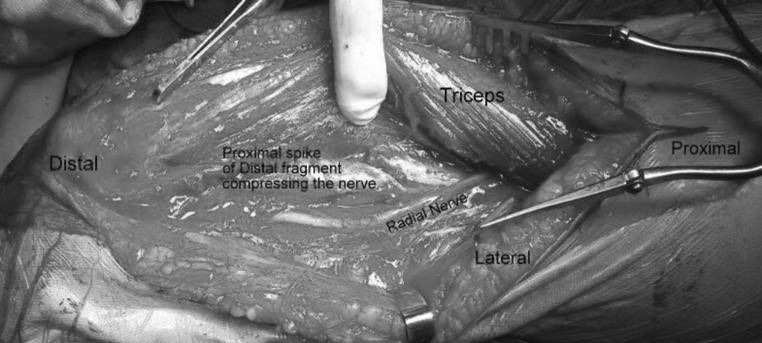
Compression of the radial nerve by proximal spike of the distal fragment

It was retrospective study of 20 patients with extra-articular distal humeral shaft fractures who were operated using the EADHP system from Sept 2011 to May 2014. Patients age, sex, mode of injury, interval between injury and surgery, status of radial nerve, associated injuries, time to union and elbow range of motion were noted. The final DASH score was measured at 1 year. Additional support in the form of elbow brace/plaster-of-paris cast/slab was not used in any of the patients. The average age of the patients at the time of surgery was 44 years (range 31–56 years) with 13 males and 7 females. The most common mode of injury was road traffic accidents (11 patients), followed by fall from height (9 patients) and 2 had non-union. Two patients had associated radial nerve palsy, but intra-operatively the nerve was found to be intact in both the cases and nerve function recovered with time (Fig. 8). Three patients sustained additional injuries; two had an ipsilateral radial fracture, while one had an ipsilateral tibial shaft fracture. Eighteen patients were operated within 5 days of injury, whereas the other two had non-union following conservative management and were operated at 3 and 4 month interval, respectively.

**Fig. 9:**
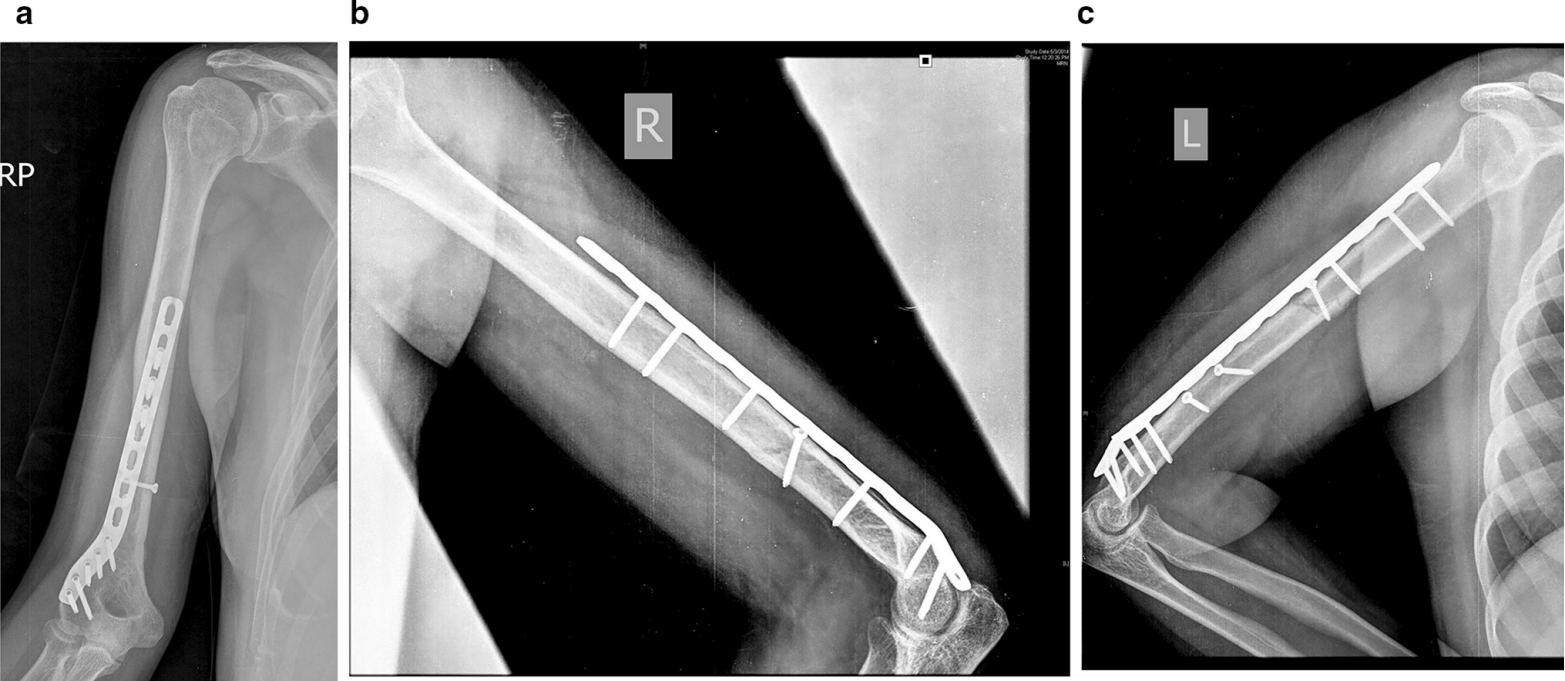
Post-op X-ray images

The mean time to radiographic fracture union was 12 weeks (range 10–18 weeks) (Fig. 9). ROM and DASH scores are presented in Table 1. At final follow-up, the mean flexion was 125° and only one patient had a flexion deformity of 5°. The mean DASH score at 1 year was 17.6 ranging from 13.3 to 38.3 points. The normal DASH score in the general population has been reported to be around 10 with a standard deviation of 14.68 [10]. There were no patients with secondary loss of reduction at the fracture site, non-union, ulnar nerve problems, superficial or deep infection. The most common fracture pattern was spiral: AO type 12 A1 (simple spiral): three cases; B1 (wedge spiral): nine cases; C1 (comminuted spiral): three cases. Lag screws (ranging from 1 to 5) were used in all the cases. Eight hole plate length was used in the majority of the cases (18 out of 20), and in the rest ten hole plate was used. A total of 3–4 screws were used for proximal fixation, and 5–6 were used for distal fixation (Fig. 9).

## Discussion

Open reduction and internal fixation of distal humeral shaft fractures is increasingly becoming an acceptable treatment modality. [5, 11–14] Options for internal fixation include intramedullary nailing and plate osteosynthesis either with double-column plating or a single column plate applied on the posterior or posterolateral side. Biomechanical studies have shown superior bending properties of humeral fractures fixed with a plate and screw system versus intramedullary devices. Also, the distal fragment is short and the medullary canal is narrow, rendering it difficult to perform nail osteosynthesis in distal third fractures [15].

Dual plating although offers a better biomechanical strength [16] does so at the expense of greater soft tissue dissection. It requires almost circumferential exposure of both the medial and lateral column. Such an enormous soft tissue dissection and exposure although justifiable for intra-articular fractures seems unreasonable for extra-articular shaft fractures. Preservation of the soft tissue envelope is an important aspect in fracture healing, and it has led to the change in the earlier concept of anatomic reduction and rigid fixation [17]. This concept is no longer valid for most of the extra-articular fractures with complex fracture patterns, where minimal soft tissue dissection and stable fixation has shown to have better results and is now the standard principle [18]. Although there have been no comparative studies of dual column vs. single column fixation for distal humerus fractures, we believe and suggest that the higher infection and non-union rates quoted in many series of distal humerus fractures may in part be due to greater soft tissue dissection and a longer operative time required for dual column plating [5, 8].

Yang et al. [18] also suggested that the excessive soft tissue dissection required for dual plating may be responsible for the increased incidence of iatrogenic radial nerve palsy reported in some series. Placement of implant over the distal medial aspect of humerus which has a scant soft tissue cover also leads to a high incidence of implant-related complications such as ulnar neuropathy [19]. To circumvent these problems, single column plating has been suggested by many to be the answer. Standard single column plating techniques fail to achieve adequate stabilization owing to many factors; the most important being inadequate distal purchase. Levy et al. [20] used modified Synthes Lateral Tibial Head Buttress Plate (Synthes, Paoli, PA) that allowed for a centrally placed posterior plating of the humeral shaft that angled anatomically along the lateral column to treat far distal humeral shaft fractures.

The advent of modern locking plates has allowed improved fixation of the peri-articular fractures. Numerous studies have demonstrated and confirmed the increased stability provided by locking plates at the distal femur, proximal tibia, calcaneum, distal radius and proximal humerus [21–25]. This increased strength of fixation has in some cases obviated the need for dual column fixation. Several studies have demonstrated that the mechanical stability and overall stiffness of a laterally placed locked plate in the proximal tibia is equivalent to the control of historical dual plating [26–28].

The extra-articular distal humeral locking plate is based on a similar concept of single column plating. Owing to greater screw hole density distally, it allows the placement of adequate number of screws in the distal fragment and the locking construct increases the stability. Since only the lateral column is exposed, it decreases both the soft tissue dissection and the surgical time. As compared to the trochlea, the posterior aspect of the lateral column is non-articular and allows for posterior placement of implant without risk of injury to the cartilage or risk of impingement with flexion and extension. We in the present series used the posterolateral approach of Gerwin et al. [9] which has several advantages over the traditional triceps splitting approach. Sparing the triceps muscle limits the formation of intramuscular adhesions and scar formation and theoretically reduces the chances of elbow contracture and improves post-operative triceps function. The exposure can be extended proximally and distally; proximal extension is by elevating the triceps off the humerus and mobilizing the radial nerve, and distal extension can be accomplished by converting the approach into an olecranon osteotomy approach, TRAP approach [29] or Bryan and Morrey [30] approach if there is an intra-articular extension of the shaft fracture. Triceps-reflecting anconeus pedicle (TRAP) approach involves complete detachment of triceps from proximal ulna along with anconeus using sharp dissection. The entire flap is then lifted off the posterior aspect of distal humerus. Lewisky, Sheppard and Ruth described how the posterolateral approach can be extended proximally and distally to expose most of the posterior humeral shaft and elbow joint for complex fracture treatment. They described the combined olecranon osteotomy, lateral paratricipital sparing and deltoid insertion splitting (COLD) approach [31]. Approximately 94% of the humeral diaphysis can be exposed with the posterolateral approach (Fig. 6c) as compared to the triceps splitting approach which provides exposure to only 76% of the shaft [9]. This enhanced exposure also provides complete visualization of the radial nerve on both sides of the inter-muscular septum and since it exploits a relatively blood less plane, this approach can be performed without a tourniquet.

DASH score was used to assess the functional outcome. This questionnaire asks the patient about symptoms as well as their ability to perform certain activities. The questions are answered based on the condition in the last week. If patient did not have an opportunity to perform an activity in the last week, the best estimate is made. It does not matter which hand or arm is use to perform the activity. The normal DASH score in the general population has been reported to be around 10 with a standard deviation of 14.68 [10].

Our study has a few limitations, namely a small sample size, and the lack of a biomechanical study to test and compare the strength of a single column vs. double-column locking plate.

As the plate is pre-contoured, it does not seat equally well in all patients and bending the plate can potentially damage the locking hole screw threads and can also change the screw direction to a certain extent. Improperly locked screws can compromise the stability of the construct, and the change in screw direction can pose a problem in the distal screws which are directed into the capitellum and trochlea. To circumvent this problem, plate bending should be done after blocking the screw holes with locking sleeves and bending the plate only in between the screw holes.

Tejwani et al. [16] in their laboratory study demonstrated that a double plating construct is stiffer than one single-locking plate, especially in varus stress when the medial column is absent. We, however, in our series of 20 patients did not encounter any patient with a comminuted medial column; those who had so, also had some intra-articular extension of the fracture and were treated by conventional dual plating system. The increased stress placed on a single (lateral) column fixation in the absence or comminution of the other (medial) column leads to increased strain over the implant at the fracture site, which can lead to implant failure in absence of union. This can to some extent be negated by using a longer plate with widely spaced screws to increase the working length.

## Conclusion

The EADHP system using the modified posterior approach to the humerus is a useful treatment option for managing extra-articular distal humerus fractures. The provision of greater screw hole density of the plate distally and using 3.5-mm screws instead of 4.5 mm allows adequate number of screws to be placed in the distal fragment. Bi-columnar fixation of distal humerus provides increased stability, but requires increased soft tissue dissection. EADHP fixation of distal humerus fractures using the modified posterior approach provides stable fracture fixation with adequate exposure of the radial nerve and >90% of posterior humeral shaft surface.
